# Microsatellite DNA typing for assessment of genetic variability in Marwari breed of Indian goat

**DOI:** 10.14202/vetworld.2015.848-854

**Published:** 2015-07-12

**Authors:** Anoop Singh Yadav, Kritika Gahlot, Gyan Chand Gahlot, Mohd Asraf, Mohan Lal Yadav

**Affiliations:** Department of Animal Breeding & Genetics, College of Veterinary & Animal Science, Rajasthan University of Veterinary & Animal Science, Bikaner - 334 003, Rajasthan, India

**Keywords:** allelic frequency, heterozygosity, Marwari goats, microsatellite marker, polymorphism information content

## Abstract

**Aim:**

To estimate existing within-breed genetic variability in Marwari goats under field conditions and the generated data that can be used to determine genetic relationships with other breed of goats.

**Materials and Methods:**

A total of 146 blood samples of goats of Marwari breed were randomly collected from genetically unrelated animals from different villages of Bikaner Districts of Rajasthan, India. Genomic DNA was extracted from whole blood using proteinase K-digestion followed by standard phenol–chloroform extraction procedure at room temperature and confirmed through horizontal electrophoresis on 0.8% agarose gel containing ethidium bromide. Fifteen caprine microsatellite markers were used to estimate genetic variability among the goats of Marwari breed in terms of allelic and genotype frequencies, heterozygosities and polymorphism information content (PIC) value.

**Results:**

A total of 74 alleles were contributed by Marwari goat across all 15 microsatellite loci. The number of alleles per locus varied from two (ILSTS-087) to 9 (ILSTS-058) alleles, with a mean of 4.93 whereas the effective number of allele varied from 1.35 (ILSTS-005) to 3.129 (ILSTS011) with a mean of 2.36. The effective number of allele is lesser than observed number at all the loci. Allelic sizes ranged from 125 bp (ILSTS-028 and ILSTS-033) to 650 bp (ILSTS-011 and ILSTS-019). The expected heterozygosity ranged from 0.240 (locus ILSTS-005) to 0.681 (locus ILSTS-011), with an average value of 0.544. The observed heterozygosity (Ho) ranged from 0.1428 (locus ILSTS-087) to 0.9285 (locus ILSTS-034), with an average value of 0.5485 indicates substantial and very good number of heterozygotes, in the population. The highest PIC value (1.1886) was observed at ILSTS-044 locus and least (0.0768) at ILSTS-065 locus for Marwari goat.

**Conclusion:**

Microsatellite analysis revealed a high level of polymorphism across studied microsatellite markers and informativeness of the markers for genetic diversity analysis studies in Marwari goats. This high level of polymorphism can be utilized to plan future biodiversity studies to exploit the uniqueness and adaptability of this breed to Western Rajasthan. Most studied microsatellite markers proving to be good candidates for genetic characterization and diversity analysis of this breed of goat.

## Introduction

Marwari goat, a major meat breed of Rajasthan (India), is well adapted to the arid environment, grows faster, bred efficiently, can tolerate higher salt loads, and requires less water than many other species of this region [1]. These unique characteristics of this breed require its molecular characterization, genetic differentiation and relationships with other breeds.

Among the various molecular marker systems for genetic characterization, microsatellites markers have been widely used as genetic markers in bovine population studies and pedigree verification, mainly because of their large polymorphism information content (PIC), widespread distribution in the eukaryotic genome (Tautz and Renz, 1984) and robust methodology. Microsatellites have been effective in evaluating differences within cattle breeds and in determining population substructures [2]. More than 1400 microsatellites have been mapped in the cattle genome [3]. There are close similarities between cattle, sheep and goat chromosomes [4]. Microsatellite markers present in all three species could be amplified with the same primer pairs, so microsatellite markers developed in cattle and sheep also work in goats [5] and they can be used for the analysis of genetic diversity [6].

Most of the studies using microsatellites have concentrated on cattle, sheep and pigs while the information available about the genetic characterization of goats is limited [7]. Therefore, this study was undertaken to analyses existing within-breed genetic variability in Marwari goat and the feasibility of generated data to determine the genetic relationship with other breed of goats.

## Materials and Methods

### Ethical approval

All essential procedures of sample collection were performed strictly as specified by Institutional Ethics Committee with minimal stress to animals.

### Location of study

The study was conducted at the College of Veterinary and Animal Science, Rajasthan University of Veterinary and Animal Science, Bikaner, Rajasthan, India, located at 27°29′ North latitude and 77°40′ East latitude (ms l-174 m). The climate of the study area is classified as tropical (semiarid zone). The temperature in this region varies from 49°C (May and June) to −2°C (winter). Low and erratic rainfall (39-392 mm) is a common feature. The soil is sandy, and vegetation is composed of natural pasture and bushes.

### Flock description and management

The Marwari goat, a native breed of arid and semi-arid desert region of West Rajasthan, is predominantly found in Jodhpur, Pali, Nagour and Bikaner districts of West Rajasthan. They are primarily used for meat purpose. Goats were allowed free range grazing on the natural pasture from 08.00-17.00 h daily except during the summer (April to June) when split grazing during cooler hours of the day was observed from 06.00 to 12.00 and 15.00 to 19.00 h. Supplementary feeding to pregnant and lactating ewes and young lambs in the form of a concentrate and harvested fodder were provided.

### Sampling and DNA isolation

Blood samples were randomly collected from 146 genetically unrelated animals of Marwari goat from different villages of Bikaner district of Rajasthan in line with MoDAD recommendations [8]. Genomic DNA was extracted from whole blood using proteinase K-digestion, followed by standard phenol–chloroform extraction procedure at room temperature [9] with few modifications. All DNA samples were analyzed for qualities on 0.8% agarose gel through horizontal electrophoresis.

### Microsatellite markers

A panel of 15 microsatellite markers was selected from the list as recommended by International Society for Animal Genetics and FAO’s (DAD-IS) for Caprine, based on their level of polymorphism, allele size range and reliability of allele calling to characterized and reveal the extent of genetic diversity in Marwari goats as follow:

### Polymerase chain reaction (PCR)-based microsatellite DNA typing

PCR was carried out in 50 μl reaction volume containing 1.5 mM MgCl_2_, 200 μM dNTPs, 1.0 μl of each primer, ~3.0 μl of template DNA and 0.25 μl of Taq DNA polymerase (Promega, Madison, USA) using PX-2 Thermocycler (Thermo Fisher, USA). PCR cycling conditions were: 5 min at 94°C, followed by 30 cycles of 1 min at 94°C, 1 min at annealing temperature (52–58°C) of each primer, 45 s at 72°C, and final extension of 30 s at 72°C. Gradient PCR was attempted to determine the exact annealing temperature. Annealing temperatures as mentioned in literature and optimized for the present study are given in Table-1. No significant change was observed by varying MgCl_2_ concentration; hence 1.5 mM concentration already present in the assay buffer was used for all amplifications. Taq DNA polymerase was initially used at 5 U (Bangalore, Genei) but later reduced to 1.25 U (Promega, USA) per reaction.

**Table-1:** Details of microsatellite markers used in goats of Marwari breed.

Locus	Primer sequence	Type of repeat	Size range	Chromosome number	Annealing Temperature (in °C)
ETH-152	TACTCGTAGGGCAGGCTGCCTG GAGACCTCAGGGTTGGTGATCAG	(CA)_17_	92-122	05	56.0
ETH-225	GATCACCTTGCCACTATTTCCT ACATGACAGCCAAGCTGCTACT	(CA)_18_	146-160	14	54.0
ILSTS-005	GGAAGCAATGAAATCTATAGC CTGTTCTGTGAGTTTGTAAGC	(nn)_39_	174-190	10	52.8
ILSTS-011	GCTTGCTACATGGAAAGT GC CTA AAATGC AGA GCC CTA CC	(CA)_11_	167-173	14	54.0
ILSTS-019	AAGGGACCTCATGTAGAAGC ACTTTTGGACCCTGTAGTGC	(TG)_10_	142-162	Ann	53.7
ILSTS-022	AGTCTGAAGGCCTGAGAACCC TTACAGTCCTTGGGGTTGC	(GT)_21_	186-202	Ann	55.0
ILSTS-028	TCC AGA TTTTGTACC AGA CC GTCATGTCATACCTTTGA GC	(CA)_7_	132-150	11	50.4
ILSTS-030	CTGCAGTTCTGCATATGTGG CTTAGACAACAGGGGTTTGG	(CA)_13_	159-179	2	54.0
ILSTS-033	TATTAGAGTGGCTCAGTGCC ATGCAGACAGTTTTAGAGGG	(CA)_12_	151-187	12	54.6
ILSTS-034	AAGGGTCTAAGTCCACTGGC GACCTGGTTTAGCAGAGAGC	(GT)_29_	153-185	5	51.0
ILSTS-044	AGTCACCCAAAAGTAACTGG ACA TGTTGT ATT CCAAGT GC	(GT)_20_	142-170	Ann	50.0
ILSTS-058	GCCTTACTACCATTTCCAGC CATCCTGACTTTGGCTGTGG	(GT)_15_	136-188	17	54.0
ILSTS-059	GCTGAACAATGTGATATGTTCAGGGGGAC AATACTGTCTTAGATGCTGC	(CA)_4_ (GT)_2_	105-135	13	55.0
ILSTS-065	GCTGCAAAGAGTTGAACACC AACTATTACAGGAGGCTCCC	(CA)_22_	105-135	24	53.7
ILSTS-087	AGC AGA CAT GAT GACTCA GC CTGCCTCTTTTCTTG AGA GC	(CA)_14_	110-120	28	50.0

PCR-amplified products were resolved on 6% urea polyacrylamide gel electrophoresis (PAGE) denaturing sequencing gel at 75 W (Sequi Gen GT apparatus. Bio-Rad, Hercules, USA) and visualized by silver staining. Allele sizes were estimated using a 100 bp ladder (Invitrogen Life Technologies, Carlsbad, USA). The genotype of each individual animal at 25 different loci was recorded by direct counting.

### Analysis of molecular data

Genotype of each individual animal was determined and recorded from the silver-stained gels for each microsatellite locus. Different measures of within-breed genetic variations, namely number of alleles, allele frequencies, effective number of alleles (Ne), observed heterozygosity (Ho), expected heterozygosity (He), were estimated to evaluate variability at DNA level. PIC for each locus was calculated according to Botstein *et al*. [10].

## Results and Discussion

The various parameters of genetic diversity in Marwari goat such as allele number, effective number of allele, PIC, observed and expected heterozygosity within population are furnished in Table-2.

**Table-2:** Details on microsatellite markers used, number and size of the alleles, polymorphism information content and heterozygosity in Marwari goats.

Microsatellite marker	Number of asllele		Allele size (bp)	Heterozygosity		PIC
	
	A_o_	A_e_		H_o_	H_(Exp)_	
ETH-152	4	2.515	150-200	0.4285	0.603	1.1306
ETH-225	5	2.755	140-200	0.500	0.637	1.1448
ILSTS-005	3	1.315	175-350	0.2857	0.240	0.6425
ILSTS-011	7	3.129	350-650	0.5714	0.681	0.9385
ILSTS-019	5	2.932	145-650	0.7857	0.660	1.2057
ILSTS-022	4	2.143	200-350	0.5714	0.534	0.1065
ILSTS-028	4	1.44	125-245	0.4285	0.305	0.8264
ILSTS-030	6	2.853	150-310	0.6428	0.650	1.1608
ILSTS-033	4	2.939	125-175	0.514	0.655	0.1862
ILSTS-034	6	2.68	200-250	0.9285	0.627	1.1759
ILSTS-044	5	2.995	150-550	0.6428	0.666	1.1886
ILSTS-058	9	2.652	175-550	0.714	0.623	0.1279
ILSTS-059	5	1.988	150-250	0.7857	0.497	1.1534
ILSTS-065	5	2.099	145-550	0.2857	0.524	0.0768
ILSTS-087	2	1.34	150-200	0.1428	0.254	0.6498
Average	4.93	2.385		0.5485	0.544	0.78096

PIC=Polymorphism information content

All the loci were amplified successfully and exhibited substantial levels of genetic diversity estimates (Figure- 1 and 2). A total of 74 alleles were observed and the number of alleles per locus varied from two (ILSTS-087) to nine (ILSTS-058), with a mean of 4.93, whereas the effective number of allele varied from 1.35 (ILSTS-005) to 3.129 (ILSTS011) with a mean of 2.385. The effective number of allele is lesser than observed number at all the loci. Barker *et al*. [11] suggested that loci with at least four alleles are suitable for studying the genetic diversity. The present study observed more number of alleles then recommended and supports the suitability of microsatellite marker or analyzing genetic diversity.

**Figure-1:**
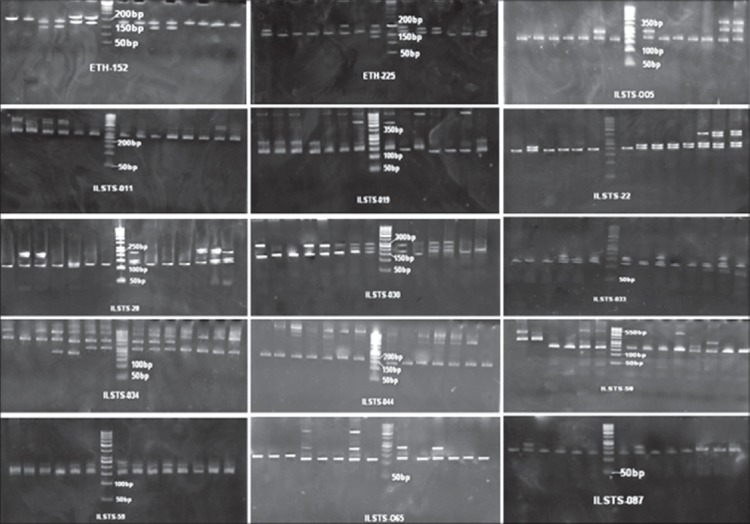
Allelic profile across the 15 microsatellite markers in Marwari goat on polyacrylamide gel electrophoresis.

**Figure-2:**
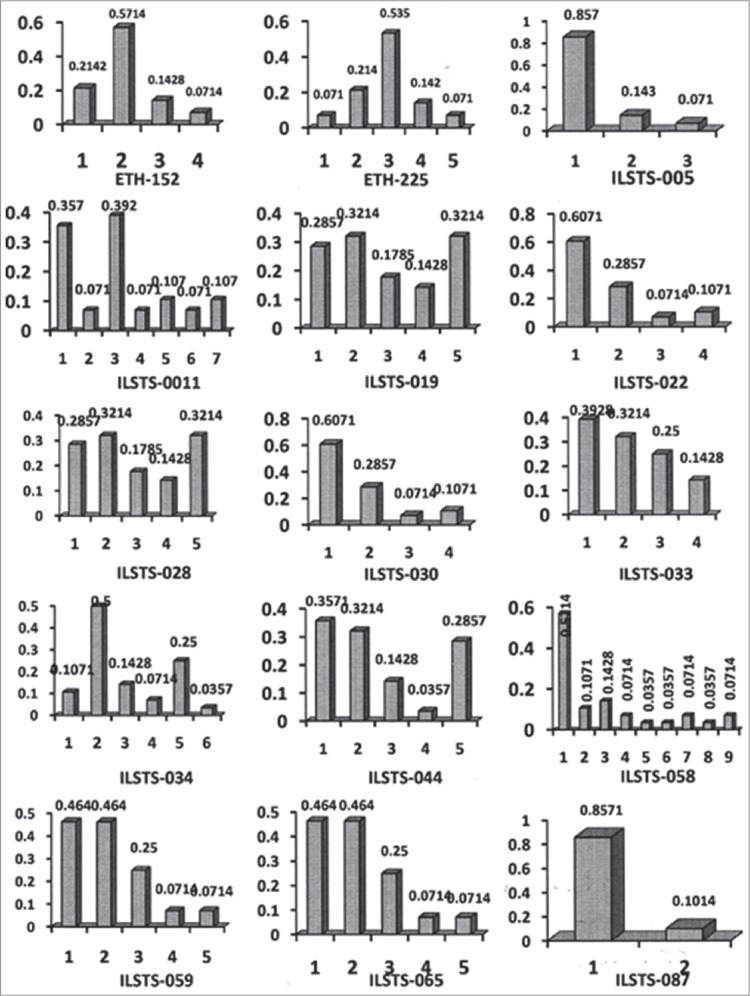
Allelic frequency distribution across the 15 microsatellite markers in Marwari goats.

The average number of observed alleles found in Marwari goats was comparable with Assam Hill goats, which ranged from 2 to 10 with an overall mean of 4.9 [12]. It was lower than that of obtained for Barbari goats (India) i.e., 6.3 or 8.1 [13,14] for Ganjam goats 6.29 [15], for Egyptian and Italian goat breeds 6.5 [16], for Raeini goats (Iran) 7.8 [8], for Jakhrana goats 9.7 [14], for Gohilwari breed of goats (Gujarat) 10.12 [17], for Berari goats (Maharashtra) 11.76 [18], for Sirohi goats 11.92 [19], for Kutchi goats 12.0 [20] and for Mehsana goats 12.28 [21].

The allele sizes ranged from 125 bp (ILSTS-028 and ILSTS-033) to 650 bp (ILSTS-011 and ILSTS-019). The number of alleles available in literature was 4 for ILSTS059, 6 for ILSTS022, ILSTS065 and ILSTS087, 7 for ILSTS019 and ILSTS028, 8 for ILSTS044 and ILSTS058 in Barbari goats from India [13]. It has been reported that there was a positive relationship between the number of di nucleotide repeats and the number of alleles at a given locus, with the number of alleles per locus ranging from one to 18 [22]. Greater the number of alleles at given locus, more informative will be the marker.

The heterozygosity is an appropriate measure of genetic variability within a population because genetic diversity can be measured as the amount of actual or potential heterozygosity. The observed heterozygosity (H_0_) ranged from 0.1428 (locus ILSTS-087) to 0.9285 (locus ILSTS-034), with an average value of 0.5485, while the expected heterozygosity ranged from 0.240 (locus ILSTS-005) to 0.681 (locus ILSTS-011), with an average value of 0.544 across the 15 microsatellite markers for the Marwari goats population.

The average genetic variation (H_0_=0.55) observed in the present study are higher than that of in Assam Hill goats, 0.43 [12] but lower than that of Black Bengal goats [23], Barberi goats [16] and other Indian breeds [24], Asian and Australian breeds [11], Swiss goats [6], and Chinese goats [7].

The expected heterozygosity (0.544) obtained in this study was higher than other studies in Barberi goats, 0.6208-0.8509 [13], and Assam hill goats 0.48 [12] but lower than for Chinese goats 0.671 [7]. The higher heterozygosity values observed has resulted in instability of the population at the majority of microsatellite loci studied. Because of higher heterozygosity and consequent non-fixation of alleles at these loci, there is further scope for improvement of the breed.

Locus ILSTS-034 exhibits the highest level of observed heterozygosity and ILSTS-087 locus exhibits the lowest observed heterozygosity. The low observed heterozygosity 0.1428 (ILSTS-087) was observed in the present study may be due to the presence of more homozygote individual in the samples analyzed. Though few loci exhibited lower heterozygosity values, most of the loci showed relatively higher expected heterozygosity, which reflects the existence of differentiation in the population. The locus with the highest level of heterozygosity is the most informative locus for Marwari goat breed. The Chi-square (*χ*^2^) test revealed that 15 microsatellite loci in the Marwari goat population are in equilibrium. These results established that the samples were drawn from the large random mating population.

The statistical assessment of the informativeness of a marker, denoted by the PIC value, varied as low as 0.0768 (ILSTS-065) to as high as 1.2057 (ILSTS-019) for polymorphic markers with mean PIC of 0.78096, which is regarded slightly informative (<0.5). Reported PIC values for these markers in other goat breeds have shown that they are well suited for genetic diversity analysis in goats [25,26]. However, it is difficult to compare among studies because some of these studies have only tested different marker sets have been used, and some may not have reported monomorphic loci.

The average value (0.78096) of PIC estimated in the present study are comparable with those values obtained in Chinese goat breeds, which ranged from 0.746 to 0.800 [7], in Croatian Spotted goats (0.743) [16] in Tali goats (0.704) and Raeini goats (0.778) [8], Lori goats (0.725) and in Markhoz goats from Iran were 0.767 [27]. In contrast, lower PIC values were obtained for Korean (0.350), Chinese (0.620) and Saanen (0.570) goats [28] and for Sirohi, Jamnapari and Barbari (0.48) Indian goats using cattle microsatellite markers [29]. The higher PIC value in the Marwari goats indicates that higher genetic diversity and subsequently low levels of inbreeding. The significant level of variability in this population reflects that the Marwari population contains a valuable genetic diversity. Hence, this population could provide a valuable source of genetic material that may be used for meeting the demands of future breeding programs.

## Conclusion

Microsatellite analysis revealed a high level of polymorphism across studied microsatellite markers and informativeness of the markers for genetic diversity analysis studies in Marwari goats. This high level of polymorphism can be utilized to plan future biodiversity studies to exploit the uniqueness and adaptability of this breed to Western Rajasthan. Based on the PIC values, the microsatellite primers used in the present study are proved to be highly polymorphic in nature and hence can be well utilized for molecular characterization of Marwari goat germplasm.

## Author’s Contributions

ASY, KG designed the work plan, collected, processed the blood samples, carried out PCR and electrophoresis. MA helps to carried out PCR and PAGE electrophoresis. GCG and ASY compiled, tabulated, transformed and analyzed the data. MA and GCG interpreted the results. ASY, KG and MLY prepared the manuscript. All authors read and approved the final manuscript.
